# Using a Y‐Shaped Artificial Ligament for Posterolateral Corner Reconstruction in Multiligament Knee Injuries: A Surgical Technique

**DOI:** 10.1002/atn2.70333

**Published:** 2026-10-29

**Authors:** Hecheng Zhou, Zhe Xue, DingRan Wang, ShiJin Xu, Jin Zhang

**Affiliations:** ^1^ Department of Sports Medicine, Beijing Jishuitan Hospital Capital Medical University Beijing P. R. China; ^2^ Department of Sports Injury and Arthroscopic Surgery National Institute of Sports Medicine Beijing P. R. China

## Abstract

In multiligament knee injuries, reconstruction of the posterolateral corner is essential for restoring varus and rotational stability of the knee. Autografts and allografts are the current mainstays for posterolateral corner reconstruction; however, their use is associated with inherent limitations, including graft availability, insufficient size, and donor site morbidity. This technical note describes a surgical technique for posterolateral corner reconstruction using a Y‐shaped artificial ligament. This approach achieves anatomic reconstruction of the lateral collateral ligament and a functional, nonanatomic reconstruction of the popliteus tendon through a single femoral tunnel. The technique aims to provide reliable knee stability in a relatively minimally invasive manner while circumventing the limitations associated with traditional grafts.

## Graphical abstract

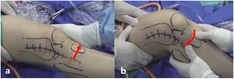

**VIDEO 1:** Surgical technique for Y‐shaped artificial ligament reconstruction of the posterolateral corner. The patient is positioned supine with the right limb secured in a foot holder with the knee flexed to 90°. This video shows:1.A standard open posterolateral corner reconstruction approach, including skin incision, soft‐tissue exposure, and identification of key anatomic landmarks.2.Selection of a single femoral tunnel for the Y‐shaped ligament.3.Configuration of the Y‐shaped artificial ligament, showing its 3 limbs (7.5‐mm trunk, 6‐mm lateral collateral ligament limb, and 4‐mm popliteus tendon limb) and their respective target structures.4.Graft passage through the bone tunnels and the sequence of fixation with interference screws. Video content can be viewed at https://doi.org/10.1002/atn2.70333.

Multiligament knee injuries (MLKIs) are defined as partial or complete tears involving at least 2 of the major ligamentous structures of the knee—the anterior cruciate ligament (ACL), posterior cruciate ligament (PCL), posteromedial corner (PMC), or posterolateral corner (PLC).^1^ Although MLKIs are relatively rare, accounting for approximately 0.02% to 0.2% of all orthopaedic injuries,^2^ they are typically caused by high‐energy trauma and are often accompanied by meniscal tears, neurovascular injuries, or intra‐articular fractures. The complexity and high heterogeneity of these injury patterns present significant challenges for clinical diagnosis and treatment.^3^ MLKIs result in severe knee instability and functional impairment, necessitating surgical reconstruction of all injured ligaments. Notably, the PLC is a complex structure composed of multiple ligamentous components, with the primary stabilizers including the lateral collateral ligament (LCL), the popliteofibular ligament (PFL), and the popliteus tendon (PT). These structures function synergistically to maintain the varus and external rotatory stability of the knee. Surgical reconstruction addressing only one of these components, without recognizing injuries to other structures within the PLC, cannot guarantee optimal postoperative outcomes. Therefore, surgical treatment must simultaneously restore both varus and external rotatory stabilities of the knee. Furthermore, isolated injuries to the PLC are relatively uncommon and frequently occur in conjunction with cruciate ligament injuries. Studies have shown that the posterolateral structures function synergistically with the PCL to resist posterior tibial translation. PLC deficiency can lead to failure of PCL reconstruction, resulting in an increased incidence of postoperative residual laxity and higher failure rates of cruciate ligament grafts.^4^

In MLKIs, the reconstruction strategy for the PLC should be individualized based on the injury pattern. Currently used techniques can be categorized by the pattern of instability they address. For isolated rotatory instability, procedures such as isolated PFL reconstruction^5^, ^6^ and anatomic PT reconstruction^7^, ^8^ have been described. For combined varus and rotatory instability, a wider array of options exist. These include the Larson technique^9^ and its modification, the figure‐of‐8 reconstruction (Arciero technique),^10^ which both use suspension fixation for the LCL and PFL. Other options are combined anatomic LCL and PFL reconstruction,^11^ as well as techniques that reconstruct all 3 static stabilizers, such as the LaPrade technique^9^ or the split Achilles tendon technique.^12^, ^13^ This diversity indicates that both the choice of reconstruction technique and graft for PLC reconstruction remain controversial. Furthermore, these procedures are often complex, often requiring multiple bone tunnels and multiple grafts to reconstruct each anatomic and functional structure.

In recent years, the development of artificial ligaments has provided alternative graft options for the treatment of MLKIs. Existing studies have shown that artificial ligaments can achieve favorable subjective scores and safety profiles in MLKI treatment; however, postoperative posterior laxity remains relatively common, underscoring the necessity of robust PLC reconstruction.^14^ Previous research indicates that compared with allografts or autografts, artificial ligaments offer advantages including consistent availability, absence of donor site morbidity, and controllable mechanical properties, making them particularly suitable for cases requiring substantial graft tissue in multiligament injuries.^15^ These advantages provide a rationale for developing additional PLC reconstruction strategies in the context of MLKIs. Accordingly, this technical note describes a surgical technique for PLC reconstruction in the setting of MLKIs. The technique uses a single Y‐shaped LARS artificial ligament (PPFY‐FP80, Movmedix, Dijon, France) in an open approach to achieve anatomic reconstruction of the LCL and nonanatomic reconstruction of the PT and is performed in conjunction with arthroscopically assisted anatomic reconstruction of the ACL and PCL using 2 separate artificial ligaments.

## SURGICAL TECHNIQUE

### Preoperative Preparation

After anesthesia, a comprehensive physical examination is performed to assess the severity of knee instability (Figure 1). Indications for PLC reconstruction are a lateral joint space opening exceeding 10 mm and a side‐to‐side difference in external tibial rotation greater than 10°. Concomitant cruciate ligament injuries are addressed first using arthroscopically assisted techniques, with meniscal surgery and other intra‐articular procedures performed as indicated. The patient is positioned supine, and the operative limb is secured in a foot holder with the knee flexed to 90°. A tourniquet is applied to the proximal thigh. The artificial ligaments are prepared according to the manufacturer's guidelines. For cruciate ligament reconstruction, 2 single‐bundle artificial ligaments are used, with a 7.5‐mm bundle (AC80, Movmedix) for the ACL and a 6‐mm bundle (PC60, Movmedix) for the PCL. For PLC reconstruction, a Y‐shaped artificial ligament (PPFY‐FP80, Movmedix) is prepared, featuring a 7.5‐mm main trunk, a 6‐mm limb for the LCL, and a 4‐mm limb for the PT (Figure 2). PLC reconstruction is then performed after completing the cruciate ligament reconstruction and any other intra‐articular procedures.

**FIGURE 1:**
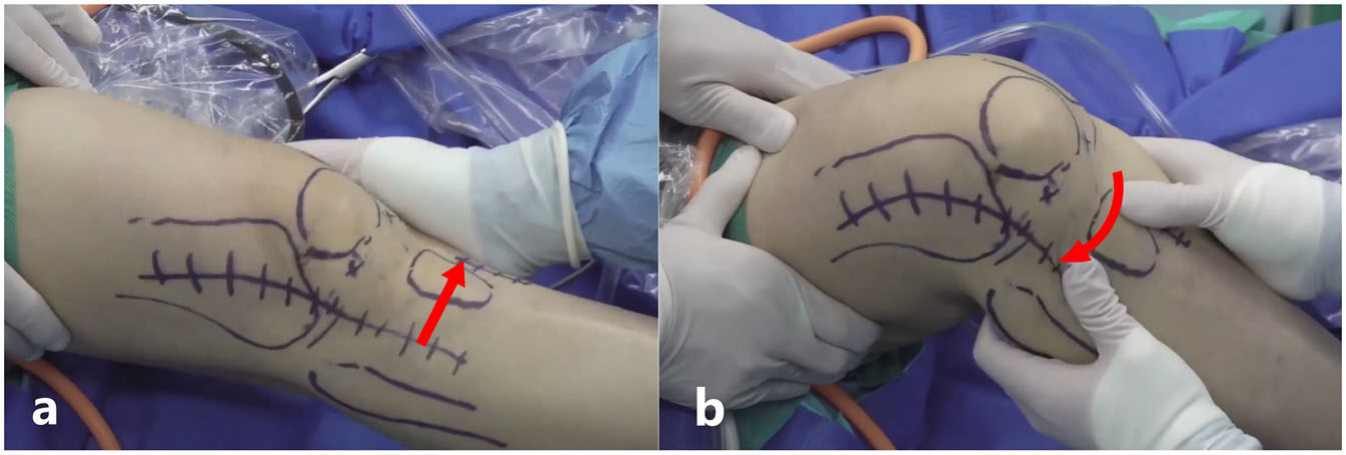
Physical examination. Clinical photograph showing a varus stress opening of >10 mm in the right knee (a), and external tibial rotation of >10° compared with the contralateral side (b). The arrow indicates the direction of force applied to the tibia during physical examination.

**FIGURE 2:**
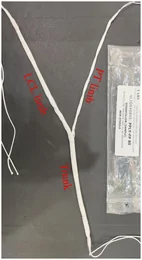
Y‐shaped artificial ligament (PPFY‐FP80, Movmedix, Dijon, France). The ligament is made of polyethylene terephthalate, and its dimensions are as indicated in the figure. (LCL, lateral collateral ligament; PT, popliteus tendon.)

### PLC Reconstruction Technique

The key steps of the procedure are shown in Video 1.

#### Tissue Exposure and Nerve Protection

A standard posterolateral approach to the knee is made. The common peroneal nerve is identified, isolated, and protected. The femoral and fibular insertions of the LCL, as well as Gerdy's tubercle, are exposed. An intermuscular tunnel is then developed for subsequent passage of the PT limb (Figure 3).

**FIGURE 3:**
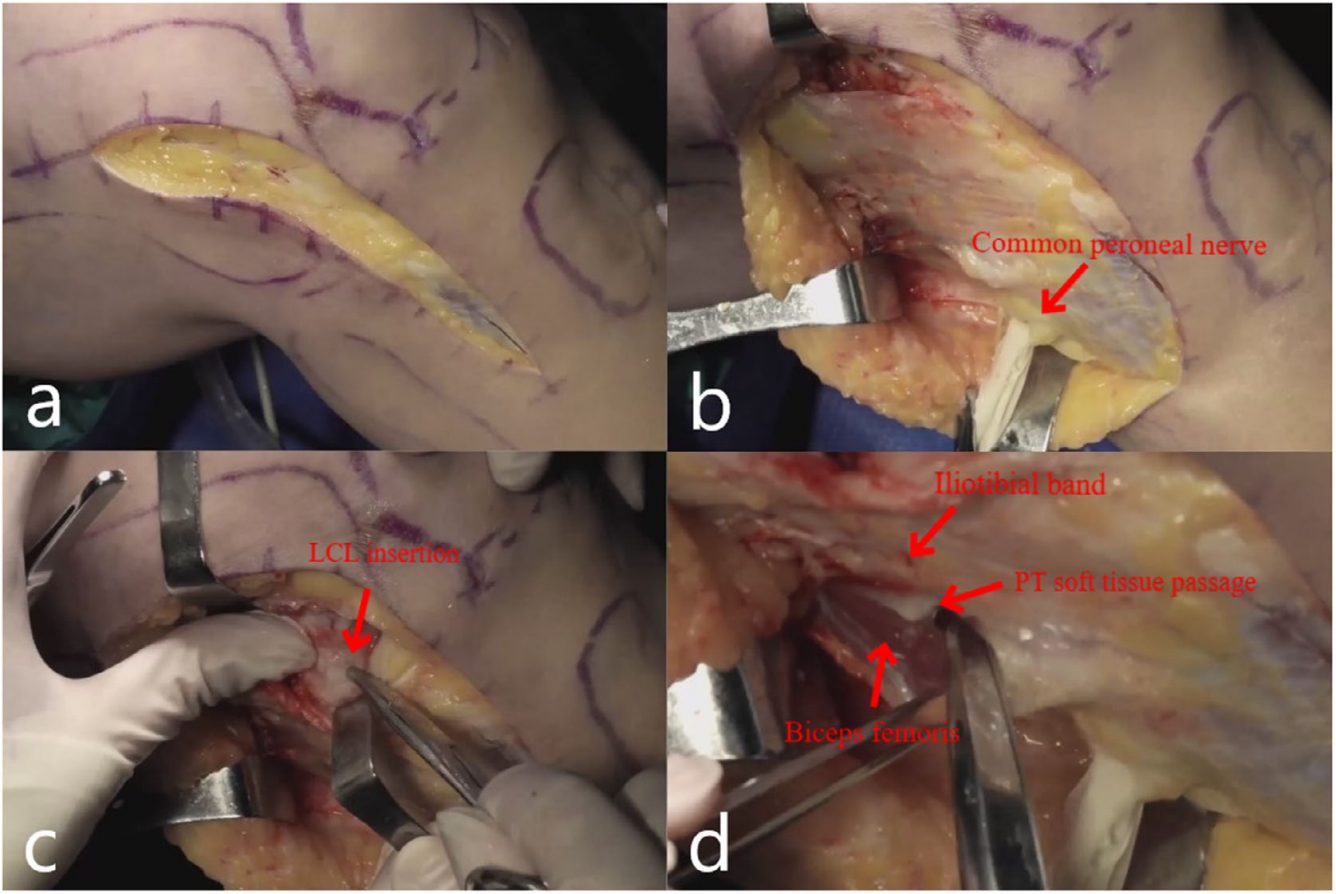
Incision and soft‐tissue dissection (right knee). The patient is positioned supine, and the operative limb is secured in a foot holder with the knee flexed to 90°. (a) A lateral skin incision is made over the knee. (b) The common peroneal nerve is identified, isolated, and protected. (c) Soft tissues are dissected to expose the anatomic insertion sites of the LCL on the anterolateral aspect of the fibular head and the most prominent point of the lateral femoral epicondyle. (d) A soft‐tissue interval is developed between the biceps femoris and the iliotibial band to prepare for passage of the popliteus tendon limb of the graft. (LCL, lateral collateral ligament; PT, popliteus tendon.)

#### Bone Tunnel Positioning and Drilling

• Fibular tunnel: A 6.0‐mm tunnel is drilled from an anteroinferior‐to‐posterosuperior direction, centered at the anatomic LCL insertion site on the fibular head (Figure 4a,b).
• Tibial tunnel: A 4.0‐mm tunnel is created between Gerdy's tubercle and the depression posterior to the tibial plateau, corresponding to the course of the PT (Figure 4c,d).
• Femoral tunnel: A single 7.5‐mm tunnel is drilled at the center of the anatomic LCL insertion site on the lateral femoral epicondyle (Figure 4e,f).

**FIGURE 4:**
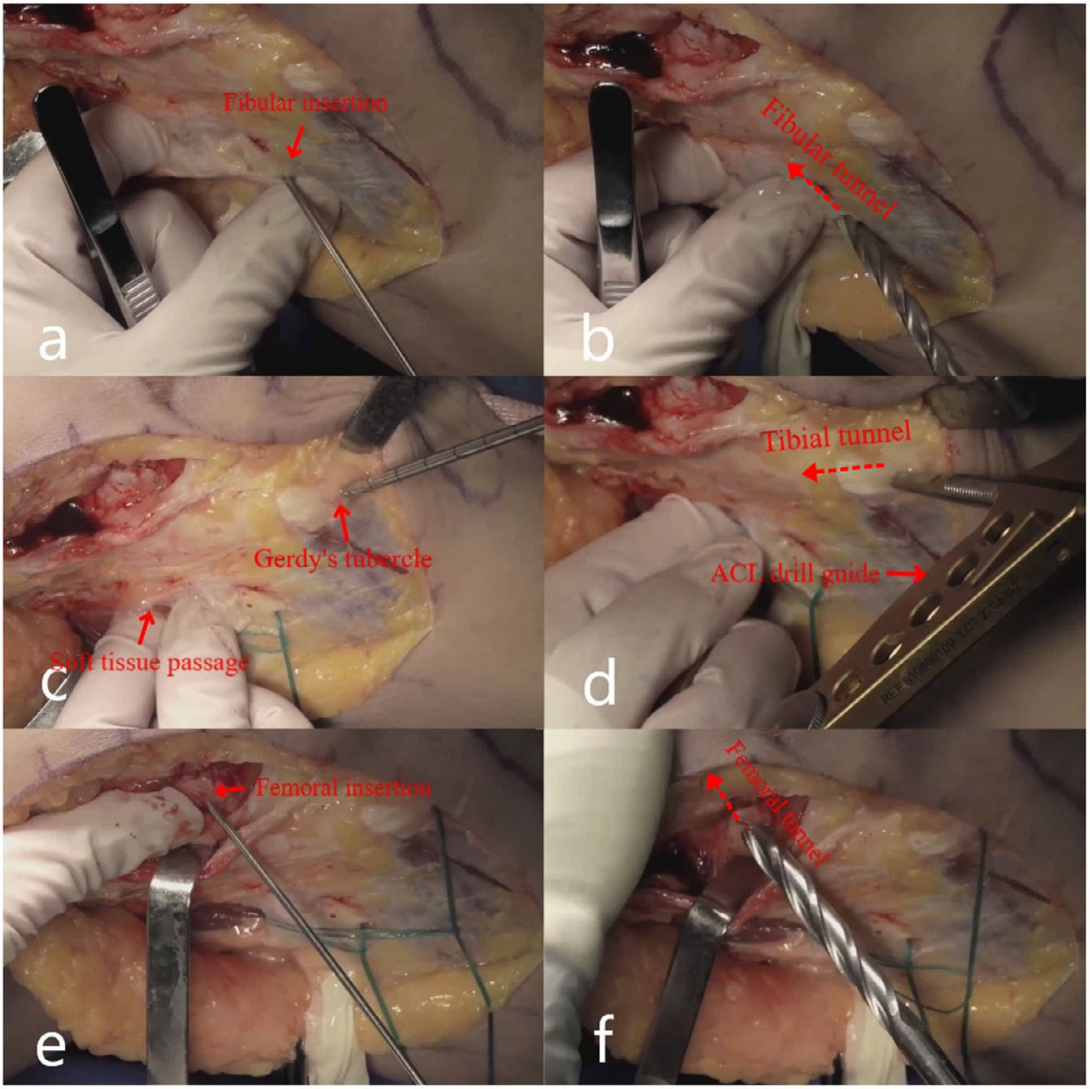
Bone tunnel positioning and drilling (right knee). (a) A K‐wire is drilled from anteroinferior to posterosuperior through the fibular head to locate the fibular tunnel for the LCL. (b) A 6‐mm drill is used to create the fibular tunnel, and a passing suture is placed. (c) For the tibial tunnel for PT reconstruction, soft tissues are dissected to identify Gerdy's tubercle. Simultaneously, a finger is inserted into the previously developed soft‐tissue interval for the PT to palpate the depression approximately 1 cm lateral to the PCL tibial insertion—the groove where the popliteus tendon courses—which serves as the tibial insertion for the PT reconstruction. An ACL guide is used to position a K‐wire for the tibial tunnel from Gerdy's tubercle to the PT tibial insertion site. (d) A 4‐mm drill is used to create the tibial tunnel, and a passing suture is placed. Care is taken to direct the drill away from the femoral tunnels for the ACL and PCL. (e) A K‐wire is positioned at the center of the LCL insertion on the lateral femoral epicondyle to locate the femoral tunnel for the Y‐shaped ligament. (f) A 7.5‐mm drill is used to create the femoral tunnel, and a passing suture is placed. The dashed arrow indicates the direction of the bone tunnel. (ACL, anterior cruciate ligament; LCL, lateral collateral ligament; PCL, posterior cruciate ligament; PT, popliteus tendon.)

#### Graft Passage, Tensioning, and Fixation

The trunk of the Y‐shaped ligament is introduced into the femoral tunnel, the LCL limb into the fibular tunnel, and the PT limb into the tibial tunnel. Fixation is then performed in a specific sequence to ensure optimal graft tension. First, the LCL limb is secured at the fibular end using an interference screw. Second, the PT limb is fixed at the tibial end. Finally, with the knee at 30° of flexion, neutral rotation of the tibia, and a gentle valgus force applied, the trunk is tensioned and secured in the femoral tunnel with an interference screw (Figures 5 and 6). This sequence ensures optimal graft tension in the functional position.

**FIGURE 5:**
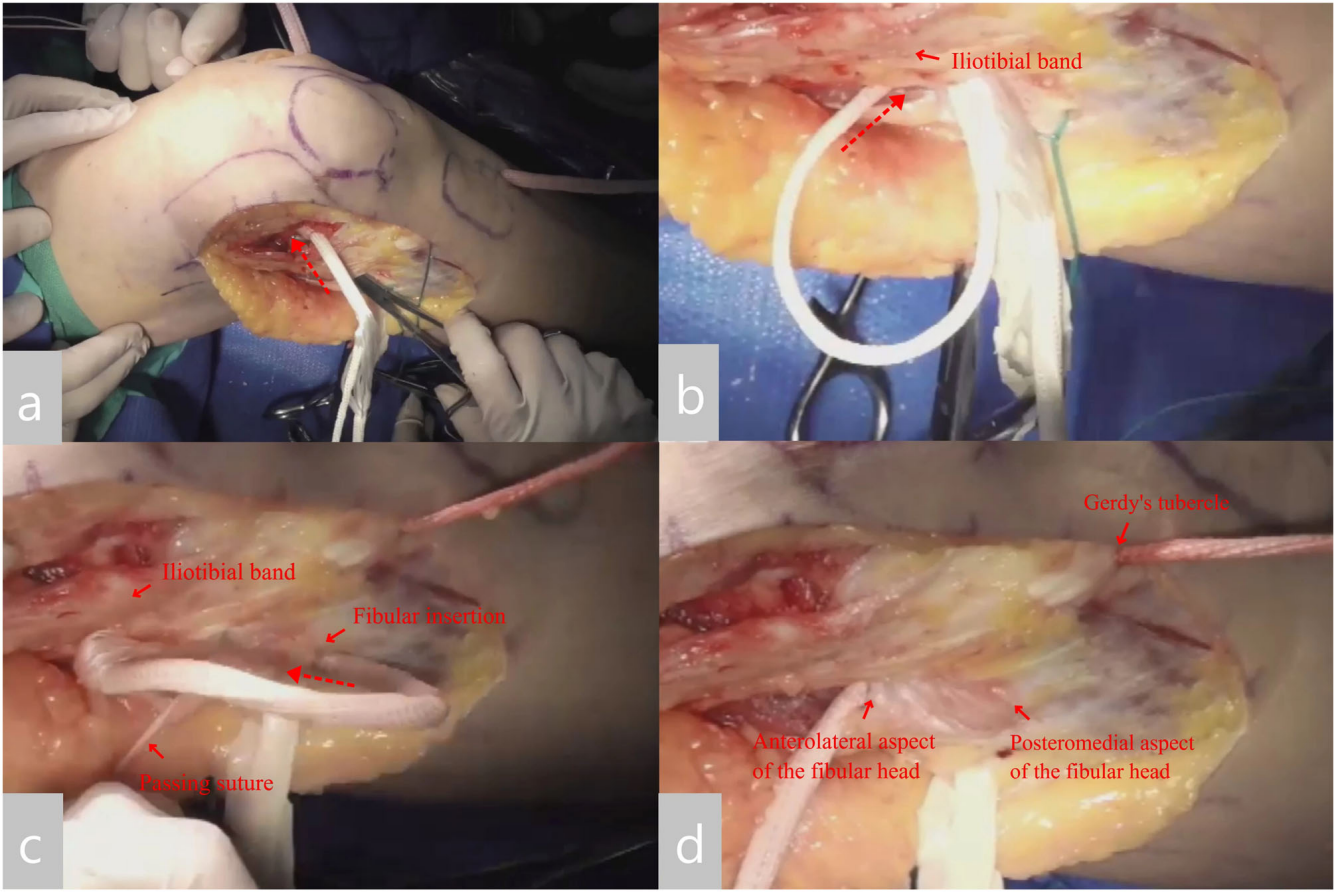
Graft passage (right knee). (a) The 7.5‐mm trunk of the Y‐shaped LARS artificial ligament is introduced into the femoral tunnel. (b) The 4‐mm PT limb is passed through a soft‐tissue channel into the tibial tunnel. (c) Deep to the iliotibial band, the 6‐mm LCL limb is introduced through the fibular tunnel in an anteroinferior‐to‐posterosuperior direction. (d) Three limbs of the graft are successfully introduced. The dashed arrow indicates the direction of graft traction. (LCL, lateral collateral ligament; PT, popliteus tendon.)

**FIGURE 6:**
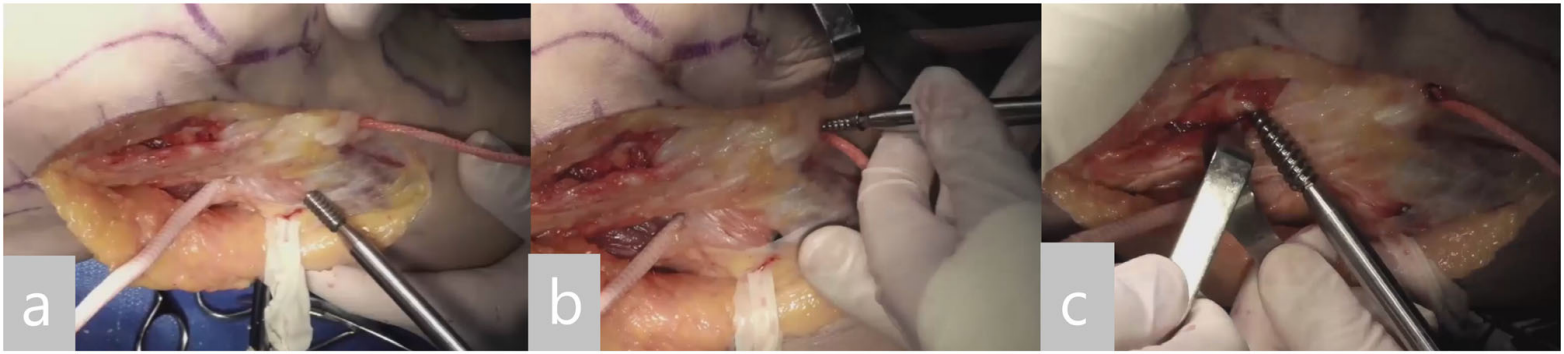
Graft fixation (right knee). (a) With the knee flexed to 90°, the LCL limb is fixed at the anterior opening of the fibular tunnel using a 7 × 25 mm interference screw. (b) The PT limb is fixed at the anterolateral opening of the tibial tunnel with a 5‐mm interference screw. (c) Finally, with the knee flexed to 30°, the tibia in neutral rotation and the knee in slight valgus, the femoral trunk of the Y‐shaped ligament is fixed at the lateral opening of the femoral tunnel using an 8 × 25 mm interference screw. (LCL, lateral collateral ligament; PT, popliteus tendon.)

### Postoperative Rehabilitation

Patients are encouraged to begin weight‐bearing ambulation on the second postoperative day. Quadriceps strengthening and passive range‐of‐motion exercises are initiated. The use of a brace is optional based on individual patient tolerance and progress. The following rehabilitation goals are targeted: full weight‐bearing ambulation and full knee range of motion by 1 month postoperatively; initiation of low‐impact activities, such as cycling, swimming, and jogging, by 3 months postoperatively; and return to full sports and daily activities by 6 months postoperatively.

## DISCUSSION

Currently, the most widely accepted PLC reconstruction techniques in clinical practice include the modified Larson technique (including the Arciero technique) and the modified anatomic LaPrade reconstruction, with the choice between them remaining somewhat controversial.^16^ Both biomechanical and clinical studies have confirmed that PLC reconstruction effectively restores varus and external rotatory stability of the knee, achieving favorable postoperative functional scores and safety profiles, with no significant differences in clinical outcomes reported between the 2 techniques.^17^, ^18^ However, the LaPrade technique is technically demanding, requiring multiple graft bundles and multiple bone tunnels, and involves extensive soft‐tissue dissection, which may increase the risk of postoperative arthrofibrosis.^17^, ^18^ With the advancement of arthroscopic techniques, short‐term evidence has tended to favor the less invasive modified Larson technique, which appears to achieve similar clinical efficacy.^19^ It is worth noting, however, that this technique is a fibula‐based reconstruction that primarily addresses the LCL and PFL, without addressing the PT. Studies by Feng and Zhang et al. have shown that isolated PT reconstruction can effectively restore external rotatory stability of the knee.^6^, ^8^ This suggests that for PLC injuries, either anatomic reconstruction or isolated reconstruction of the PFL or PT alone may be sufficient to effectively restore external rotatory stability. Most previous studies have only reported the grade or classification of PLC injuries without documenting the status of the PT—an anatomically important structure. The potential value of PT reconstruction requires further validation through high‐quality, long‐term studies with large sample sizes. Our technique addresses this gap by emphasizing functional reconstruction of the PT.

Beyond technical considerations, graft selection represents another core feature of this technique compared with traditional approaches. Artificial ligaments possess favorable biomechanical properties, and studies have confirmed that LARS artificial ligaments achieve good clinical outcomes, low graft failure rates, and faster rehabilitation in cruciate ligament reconstruction for MLKIs.^14^, ^20^, ^21^, ^22^

In previous PT‐based PLC reconstruction techniques, Y‐shaped graft configurations have been extensively explored, most notably represented by the split Achilles tendon technique,^9^, ^13^ in which 1 limb is used for PT reconstruction and the other for figure‐of‐8 reconstruction of the LCL and PFL. Although this technique achieves anatomic reconstruction and functional restoration, allografts are associated with clinical issues such as limited availability and irradiation‐induced tissue degeneration. Autografts, in contrast, make it difficult to obtain a sufficiently robust Y‐shaped structure, and although the peroneus longus tendon can meet the morphological requirements, its important role in dynamic ankle stability limits its routine use.^23^, ^24^

The present technique employs a Y‐shaped artificial ligament, effectively circumventing the aforementioned graft‐related issues. Furthermore, the use of a single femoral tunnel offers several advantages: simplified surgical steps with fewer tunnels; avoidance of tunnel conflicts with cruciate ligament reconstruction; and reduced risk of bone bridge collapse between 2 femoral tunnels, which is particularly advantageous in patients with small femoral condyles. However, recent notes have also highlighted the importance of simplified tunnel strategies, avoidance of tunnel convergence, and incorporation of the popliteus component in complex knee ligament reconstruction.^25^, ^26^, ^27^ For patients with concomitant ACL injury, the femoral tunnel for the ACL should be drilled using an inside‐out technique, aimed slightly proximally to avoid the LCL insertion on the lateral femoral condyle as much as possible. This is more easily accomplished with artificial ligament reconstruction for the ACL because we target a high isometric point on the femoral side. Importantly, the bone tunnels should be checked under fluoroscopy to rule out any convergence. For patients with PCL injury, it is sufficient to ensure that the LCL femoral tunnel is parallel to the PCL femoral tunnel.

Because of this single‐tunnel design, the femoral insertion of the PT limb is positioned more posteriorly compared with its anatomic location, with its course lying between that of the anatomic PT and PFL. As a result, this functional PT reconstruction may more closely approximate the overall function of the PT‐PFL complex. Moreover, given the excellent mechanical properties of artificial ligaments, intraoperative and immediate postoperative physical examinations have confirmed satisfactory restoration of varus and external rotatory stability despite the nonanatomic PT reconstruction, suggesting reliable early clinical efficacy of this technique. The advantages and disadvantages of this technique are summarized in Table 1, and the pearls and pitfalls are summarized in Table 2.

**TABLE 1:** Advantages and Disadvantages of Y‐Shaped Artificial Ligament Reconstruction for the Posterolateral Corner

**Advantages**	**Disadvantages**
• Y‐shaped artificial ligament eliminates graft availability issues and donor site morbidity associated with autografts/allografts • Accelerated rehabilitation potential because of robust graft fixation • The use of a single femoral tunnel simplifies the procedure, eliminates the need for additional femoral tunnel positioning for the PFL/PT, and is particularly suitable for patients with small femoral condyles	• PT reconstruction, similar to the LaPrade technique, requires extensive soft‐tissue dissection and is technically demanding • The PT reconstruction is nonanatomic. Its isometry and long‐term failure rate compared with anatomic reconstruction require further clinical investigation • Cost of artificial ligament may be higher than traditional grafts in some settings

PFL, popliteofibular ligament; PT, popliteus tendon.

**TABLE 2:** Pearls and Pitfalls

**Pearls**	**Pitfalls**
• Confirm varus opening and increased external tibial rotation before proceeding with PLC reconstruction • Identify, mobilize, and protect the common peroneal nerve before drilling any tunnel • Confirm the 7.5‐mm trunk, 6‐mm LCL limb, and 4‐mm PT limb of the Y‐shaped LARS artificial ligament before passage • Plan tunnel trajectories carefully, particularly when ACL and PCL reconstructions are performed in the same setting • Fix the LCL limb, then the PT limb, and finally tension the common femoral trunk with the knee at 30° of flexion, neutral tibial rotation, and gentle valgus force • Reassess varus and external rotatory stability after fixation and before closure	• Inadequate preoperative and intraoperative assessment may leave residual varus or rotatory laxity • Failure to protect the common peroneal nerve may result in iatrogenic nerve injury • Incorrect graft orientation or limb assignment may compromise LCL or PT reconstruction • Tunnel convergence with cruciate ligament tunnels may compromise fixation or create bone bridge failure • Undertensioning, fixation in malrotation, or fixation at an inappropriate knee flexion angle may compromise the ligament reconstruction • Under‐recognition of PLC function or residual laxity may compromise postoperative stability

ACL, anterior cruciate ligament; LCL, lateral collateral ligament; PCL, posterior cruciate ligament; PLC, posterolateral corner; PT, popliteus tendon.

Our technique uses an artificial ligament as the sole graft for PLC reconstruction, using a Y‐shaped, nonanatomic configuration with a single femoral tunnel. In the context of MLKIs, simultaneous cruciate and PLC reconstruction using artificial ligaments not only addresses the issue of graft availability in multiligament injuries but also offers the potential for accelerated postoperative rehabilitation, thereby reducing the risk of complications such as arthrofibrosis. Prospective studies are planned to systematically evaluate the clinical outcomes and define the indications for this technique.

## DISCLOSURES

The authors (Z.X., J.Z.) declare the following financial interests/personal relationships which may be considered potential competing interests: Z.X. reports a relationship with the National Natural Science Foundation of China. J.Z. reports a relationship with the Beijing Municipal Administration of Hospitals; Beijing Medical and Health Science and Technology Promotion Center; Beijing Association for Science and Technology; Beijing Jishuitan Hospital Affiliated to Capital Medical University; Beijing Nova Program; and Science and Technology Program of XPCC. The other authors (H.Z., D.R.W., S.J.X.) declare that they have no known competing financial interests or personal relationships that could have appeared to influence the work reported in this article.
